# Platelet Counts and Patent Ductus Arteriosus in Preterm Infants: An Updated Systematic Review and Meta-Analysis

**DOI:** 10.3389/fped.2020.613766

**Published:** 2021-01-20

**Authors:** Gema González-Luis, Stefano Ghirardello, Pilar Bas-Suárez, Giacomo Cavallaro, Fabio Mosca, Ronald I Clyman, Eduardo Villamor

**Affiliations:** ^1^Department of Neonatology, Complejo Hospitalario Universitario Insular Materno-Infantil (CHUIMI) de Canarias, Las Palmas de Gran Canaria, Spain; ^2^Neonatal Intensive Care Unit, Fondazione IRCCS Ca' Granda Ospedale Maggiore Policlinico, Milan, Italy; ^3^Department of Pediatrics, Hospital Vithas Santa Catalina, Las Palmas de Gran Canaria, Spain; ^4^Department of Clinical Sciences and Community Health, Università degli Studi di Milano, Milan, Italy; ^5^Cardiovascular Research Institute, Department of Pediatrics, University of California, San Francisco, San Francisco, CA, United States; ^6^Department of Pediatrics, Maastricht University Medical Center (MUMC+), School for Oncology and Developmental Biology (GROW), Maastricht, Netherlands

**Keywords:** ductus arteriosus, platelets, meta-analysis, platelet distribution width, thrombocytopenia

## Abstract

**Background:** A meta-analysis published in 2015 showed a significant association between low platelet counts in the first day(s) of life and risk of patent ductus arteriosus (PDA). The meta-analysis pooled data from 11 studies cohorts (3,479 preterm infants).

**Objective:** To update the meta-analysis by adding new studies on the topic and including other platelet parameters different from platelet counts.

**Methods:** PubMed/Medline and Embase databases were searched. Random-effects risk ratios (RR) and differences in means (DM) and 95% confidence intervals (CI) were calculated.

**Results:** We included 31 studies (7,638 infants). Meta-analysis showed that the risk of developing any PDA was significantly associated with platelet counts<150 × 10^9^/L (11 studies, RR 1.58, 95% CI 1.28 to 1.95), and <100 x 10^9^/L (7 studies, RR 1.61, 95% CI 1.14 to 2.28), but not <50 x 10^9^/L (4 studies, RR 1.34, 95% CI 0.77 to 2.32). Risk of developing hemodynamically significant PDA (hsPDA) was significantly associated with platelet counts<150 x 10^9^/L (12 studies, RR 1.33, 95% CI 1.09 to 1.63), and <100 x 10^9^/L (7 studies, RR 1.39, 95% CI 1.06 to 1.82), but not <50 x 10^9^/L (6 studies, RR 1.24, 95% CI 0.86 to 1.79). Infants with hsPDA had significantly lower mean platelet counts (19 studies, DM 22.0 x 10^9^, 95% CI 14.9 to 29.1) and platelet mass (11 studies, DM 214.4, 95% CI 131.2 to 297.5) and significantly higher platelet distribution width (PDW, 9 studies, DM −0.53, 95% CI −1.01 to −0.05) than infants without hsPDA. Meta-analysis could not demonstrate significant differences in mean platelet volume (MPV).

**Conclusion:** Compared to the previous analysis, this updated meta-analysis included 21 additional studies that provide stronger evidence of the association between low platelet counts and PDA/hsPDA. Other platelet parameters such as platelet mass and PDW are also associated with hsPDA risk. However, the low number of platelets may be an epiphenomenon associated with the maturity and clinical stability of preterm infants rather than a contributing factor in the pathogenesis of PDA.

## Introduction

Failure of ductus arteriosus (DA) closure in preterm infants is a conundrum that neonatologists have faced for decades and whose pathophysiological and clinical implications are far from being solved (1–10). In 2010, Echtler et al. made a significant contribution to the understanding of DA pathobiology when they showed that normal number and function of platelets were key factors for mice DA closure (11). They also reported an association between low platelet counts in the first day of life and risk of developing patent DA (PDA) in a cohort of 123 infants born at 24 to 30 weeks of gestation (11). The publication of these results prompted numerous groups of neonatologists to analyze the potential association between the number of platelets at birth and the presence of PDA in their populations (12–18).

In 2015, we conducted a systematic review and meta-analysis on the topic of platelet counts in the first days of life and PDA (19). The meta-analysis pooled data from 11 study cohorts (3,479 preterm infants) and showed a significant association between PDA and platelet counts<150 × 10^9^/L as well as platelet counts <100 × 10^9^/L. However, the results were largely limited by the low robustness of the association and the high degree of statistical heterogeneity among studies. The evidence was therefore highly susceptible to being modified as more researchers published new data. The objective of the present study was to update our previous meta-analysis. Since our original report, we have located 20 additional studies that we have included in a new meta-analysis. Several studies included information about other platelet factors in addition to platelet counts. These factors have been also included in our updated meta-analysis.

## Methods

The methodology employed in this updated systematic review and meta-analysis is similar to that previously described in our 2015 report (19).

### Search

We set up a monthly electronic alert from PubMed for new articles containing the most relevant search terms of our previous meta-analysis (last update June 2020). In addition, a new search was conducted in Pubmed and Embase on April 15, 2020 and we searched the Science Citation Index and Google Scholar for articles citing our previous meta-analysis as well as the articles that were included in it.

### Inclusion Criteria and Study Selection

Two investigators (G G-L, and EV) independently evaluated studies for inclusion and any disagreements were resolved by discussion. Studies were included for analysis if satisfying all following criteria: (1) full text was available in English, Spanish, Italian, Dutch, French, German, Portuguese, Galician, or Catalan; (2) a prospective or retrospective cohort study or case-control design was used; (3) primary data were reported in a manner that could be used to measure the association between PDA and platelet counts or other platelet parameters; and (4) the study population was comprised of preterm infants. As in our previous meta-analysis the studies were divided according to the way they considered small ductal shunts (19). Studies comparing closed DA vs. small plus large PDA, were classified as reporting on “any PDA.” Studies comparing closed DA and/or small PDA vs. large PDA, were classified as reporting on “hemodynamically significant PDA” (hsPDA).

### Data Extraction and Assessment of Study Quality

Two investigators (GG-L, EV) independently extracted data on study design, demographics, rate of PDA and/or hsPDA, platelet counts, and other quantitative or qualitative platelet factors. A second group of investigators (SC, GC) checked the data extraction for completeness and accuracy. In cases in which necessary data were missing from the studies, additional information was requested from the authors. Methodological quality was assessed using the Newcastle-Ottawa Scale (NOS) for cohort or case-control studies (20).

### Statistical Analysis

Studies were combined and analyzed using comprehensive meta-analysis V 3.0 software (Biostat Inc., Englewood, NJ, USA). For dichotomous outcomes, the risk ratio (RR) with 95% confidence interval (CI) was calculated from the data provided in the studies. For continuous outcomes, the difference in means (DM) with 95% CI was calculated. When studies reported continuous variables as median and range or interquartile range, we requested the mean and standard deviation from the authors or, alternatively, we estimated them using the method of Wan et al. (21). Due to anticipated heterogeneity, summary statistics were calculated with a random-effects model. This model accounts for variability between studies as well as within studies. To identify any study that may have exerted a disproportionate influence on the summary effect, we deleted studies one at a time. Heterogeneity was assessed with the Q statistic and quantified using the *I*^2^ statistic. Publication bias was assessed only for the analyses including at least 10 studies. We used the Egger's regression test and visual analysis of funnel plots to assess publication bias. Meta-regression, using random effects (method of moments estimator), was performed to explore the following sources of heterogeneity in the association between platelet counts and PDA/hSPDA: cohort mean or median gestational age (GA) and birth weight (BW), percent males, rate of PDA/hsPDA, thrombocytopenia rate, and number of infants included in the study. Meta-regression was performed only for the analyses including at least 10 studies. A probability value of <0.05 (0.10 for heterogeneity) was considered statistically significant.

## Results

### Included Studies

The new search identified 20 articles (22–41) that were added to the 11 already included in our previous meta-analysis (19). The PRISMA flow diagram of the search process is shown in Supplementary Figure 1. The 31 studies yielded a population of 7,638 preterm infants. The main characteristics of the studies are shown in Table 1. All the studies were cohort studies and only one (43) was prospective. Each study was allocated more than six NOS stars (Supplementary Table 1). The criteria used in each study for the definition of PDA and/or hsPDA are shown in Supplementary Table 2. From the included studies, 13 (11, 14–16, 19, 23, 32, 34, 37–39, 42, 43) reported on any PDA; that is, infants with a small PDA were included by the authors in the PDA group. Twenty-four studies (11–13, 16–19, 22–36, 41) compared infants with and without hsPDA; that is, infants with a small PDA were included by the authors in the non-PDA group. Six studies (11, 16, 19, 23, 32, 34) provided data on closed DA, small PDA and hsPDA. We included these studies in both analyses of any PDA and hsPDA. Twelve studies (22–27, 29–31, 35, 40, 41) reported data on mean platelet volume (MPV). Eleven studies (22–26, 29, 30, 35, 40, 41) reported data on platelet mass, and four studies (22, 26, 30, 41) reported data on plateletcrit. Since the plateletcrit is calculated with the formula platelet count (10^4^/μl) × MPV (fL) × 10^−3^ (44) and the platelet mass is calculated with the formula platelet count (10^9^/L) × MPV (fL) (45), plateletcrit values were converted into platelet mass values by multiplying by 10^4^. Nine studies (22, 23, 25–27, 30, 35, 40, 41) reported data on platelet distribution width (PDW).

**Table 1:** Characteristics of the studies included in the meta-analysis.

**Study**	**Year**	**n**	**Country**	**GA cohort (weeks)**	**BW cohort (g)**	**% males**	**Day PDA assessment**	**%PDA**	**%hsPDA**	**Range PL assessment (days)**	**% PL <150**	**% PL <100**
Echtler (11)	2010	123	Germany	28.0	1036	48.8	3–5	70.7	23.6	1	15.4	0.0
Fujioka (14)	2011	118	Japan	27.9	1059	57.6	3–5	29.7	23.7^a^	1	16.1	6.8^a^
Shah (15)	2011	497	USA, Canada	25.8	825	52.6	3	34.0	NR	3	27.2^a^	8.7^a^
Dwarakanath^b^ (42)	2011	148	USA	26.0	733	50.0	NR	60.8	45.9^c^	3	41.2	NR
Dizdar (12)	2012	361	Turkey	28.6	1054	46.5	3–5	NR	42.7	3	23.8	5.5^a^
Sallmon (16)	2012	1350	USA	28.3	1060	48.7	4–5	56.3	26.7	1	16.4	3.4
Dani (13)	2012	163	Italy	27.1	931	49.7	1–2	NR	82.8	1	32.5	19.6^a^
Brunner (43)	2013	322	Austria	29.0	1170	50.6	2–7	50.9	47.2	1	NR	2.8^d^
Bas-Suarez (18)	2014	194	Spain	27.9	1008	55.7	2–4	NR	54.6	2	11.3	6.7
Chen (17)	2014	77	Taiwan	29.5	1153	49.4	3–6	NR	44.2	1	NR	NR
Simon^e^ (19)	2015	126	NL, Italy	28.4	1096	51.2	2–4	69.8	64.3	2	28.6	14.28
Engür (27)	2015	34	Turkey	29.8	1250	58.8	2	NR	32.3	2–5	NR	NR
Demir (25)	2016	235	Turkey	29.0	1549	54	3–5	NR	48.9	0–3	16.17	NR
Kulkarni (32)	2016	70	India	31.7	1197	51.4	3	28.6	17.4	1	50.0	24.3
Meinarde (33)	2016	88	Argentina	28.4	1112	50.0	3–7	NR	50.0	3	30.7	NR
Morawietz (34)	2016	368	Germany	28.0	1043	52.9	NR	64.1	36.9	NR	26.9	NR
Oliveira (38)	2016	328	Portugal	30.0	1231	48.8	1–3	30.2	NR	NR	19.5	NR
Olukman (35)	2016	824	Turkey	29.5	1193	45.9	3–4	NR	25.2	NR	NR	NR
Temel (22)	2017	97	Turkey	32.2	1809	48.5	3–5	NR	48.5	0–3	NR	NR
Kahvecioglu (29)	2018	60	Turkey	27.8	1024	45.0	3–4	NR	40.0	0–3	NR	NR
Bekmez (41)	2018	212	Turkey	29.0	1238	59.4	2–3	54.7	NR	2–3	NR	NR
Küçuk (31)	2018	75	Turkey	28.5	1180	56.0	0–3	NR	61.3	0–1	NR	NR
Akar (24)	2019	389	Turkey	29.5	1055	49.1	2–4	NR	45.7	0–1	NR	NR
Karabulut (30)	2019	148	Turkey	28.8	1092	45.9	1–3	NR	47.9	1–7	NR	NR
Kazanci (40)	2019	481	Turkey	28.0	1068	51.3	2–4	NR	35.1	1	NR	NR
Saldaña (39)	2019	250	Peru	NR	NR	51.6	NR	NR	34.8	NR	10.8	9.2
Demirel (26)	2020	100	Turkey	28.8	1238	NR	3–5	NR	50.0	1	NR	NR
Shekharappa (36)	2020	88	India	30.4	1335	46.6	NR	NR	82.9	NR	NR	NR
Ahmed (23)	2020	75	Egypt	31.7	NR	48.0	3	53.3	NR	2	NR	NR
Ghirardello (28)	2020	151	Italy	29.1	1098	44.4	1–3	NR	24.5	0	24.5	7.3
Kusuma (37)	2020	86	Indonesia	31.4	1535	47.7	NR	50	NR	NR	34.9	NR

a *Data not reported in the original article but obtained after request to authors*.

b *Abstract*.

c *Based on numer of patients treated with COX inhibitors*.

d *Patients with platelet count <50 × 10^9^/L excluded*.

e *Data published in the meta-analysis of Simon et al. as “CPS” group. GA, gestational age (mean or median); BW, birth weight total cohort (g, mean or median); PL, platelets (×10^9^/L); NR, data not reported; PDA, patent ductus arteriosus; hsPDA, hemodynamically significant PDA. In the study of Shah et al. all the patients received prophylactic indomethacin. In the study of Echtler et al. a non-reported number of patients received prophylactic indomethacin*.

### Meta-Analysis

#### Any PDA

**Figure 1:**
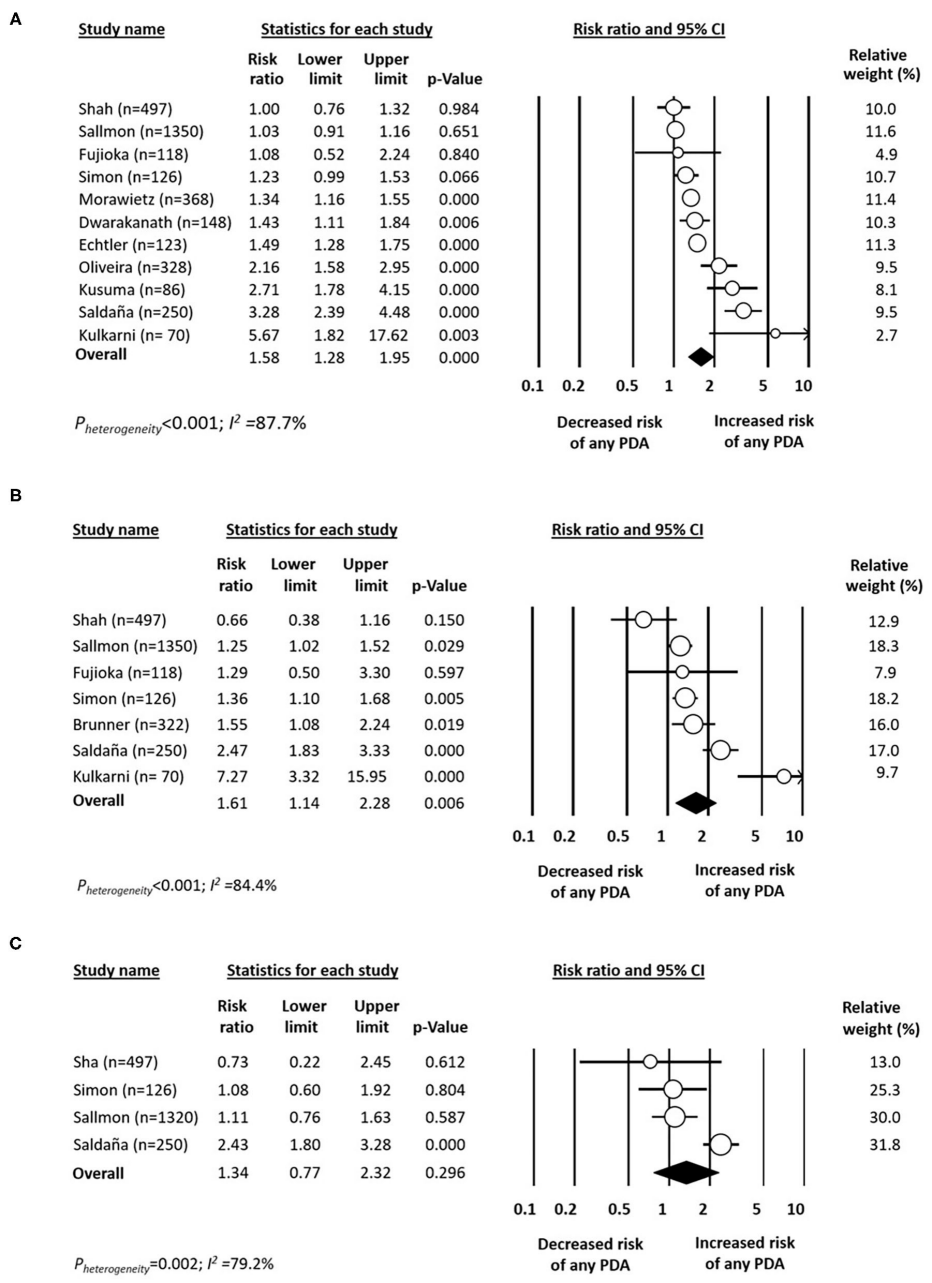
Forest plot for studies examining the association between platelet counts below **(A)** 150 × 10^9^/L, **(B)** 100 × 10^9^/L, and **(C)** 50 × 10^9^/L in the first day(s) of life on patent ductus arteriosus (PDA). Risk ratio >1 indicates increased risk of PDA.

Data on rate of any PDA in infants with platelet counts above or below 150 × 10^9^/L were available from 11 studies (11, 14–16, 19, 32, 34, 37–39, 42). There was a significant positive association between any PDA and platelet counts below 150 × 10^9^/L (Figure 1A). The results of sensitivity analyses, excluding one study at a time, for this and all other analyses are shown in Supplementary Table 3. Neither visual inspection of the funnel plot (Supplementary Figure 2A) nor Egger's regression test (*P* = 0.066) revealed evidence of significant publication bias.

**Figure 2:**
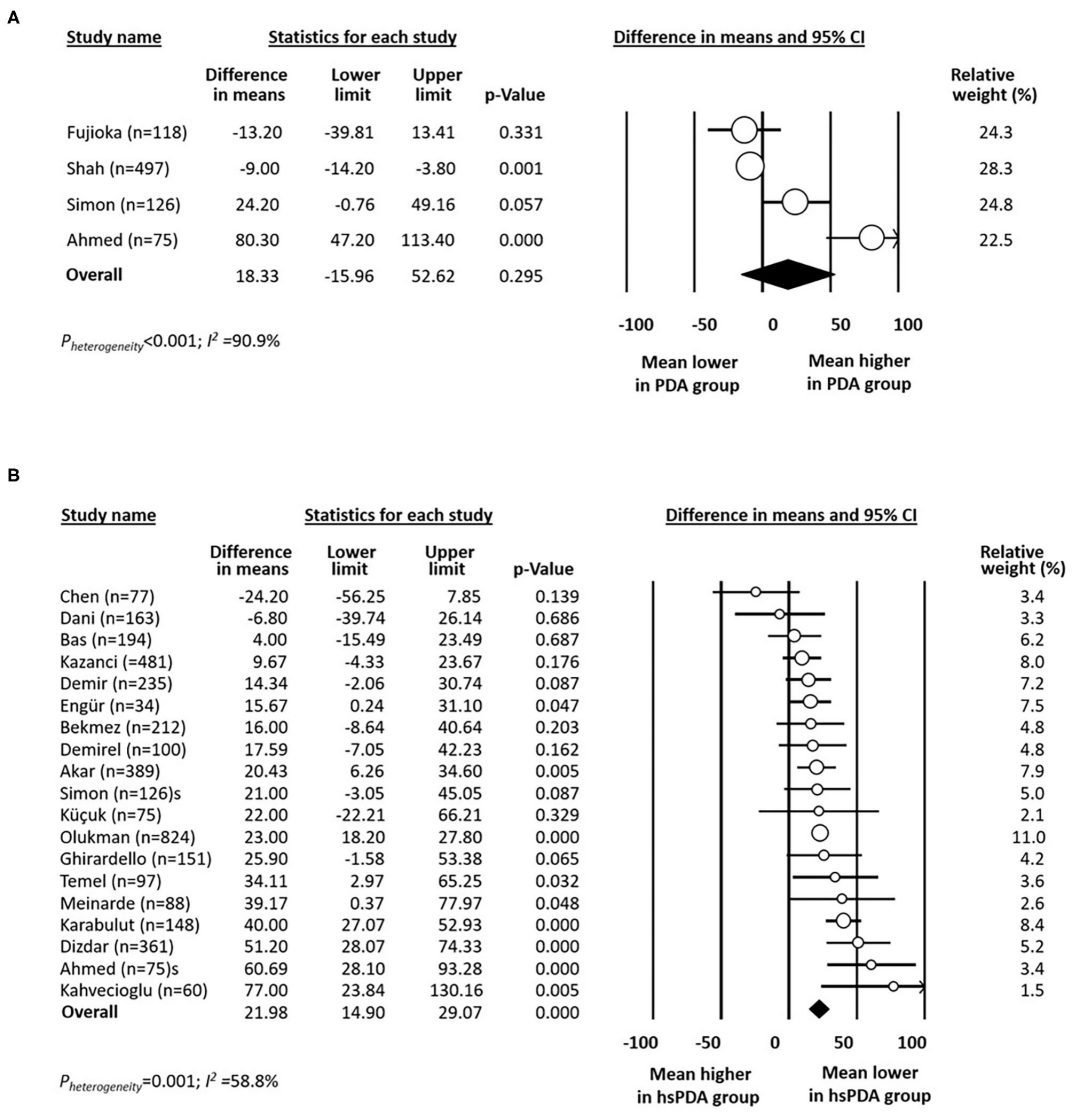
Forest plot for studies examining the difference (PDA-no minus PDA-yes) in mean platelet count (×10^9^/L) in the first day(s) of life in infants with and without any patent ductus arteriosus (PDA, **A**) and with and without hemodynamically significant patent ductus arteriosus (hsPDA, **B**).

Data on rate of PDA in infants with platelet counts above or below 100 × 10^9^/L were available from seven studies (14–16, 19, 32, 39, 43). There was a significant positive association between any PDA and platelet counts below 100 × 10^9^/L (Figure 1B). Data on rate of any PDA in infants with platelet counts above or below 50 × 10^9^/L were available from four studies (15, 16, 19, 39). Meta-analysis could not detect a significant association between any PDA and platelet counts below 50 × 10^9^/L (Figure 1C). Mean (SD) platelet counts in infants with and without any PDA were available from four studies (14, 15, 19, 23). Meta-analysis could not demonstrate a significant difference in mean platelet counts between the two groups (Figure 2A).

#### Hemodynamically Significant PDA

**Figure 3:**
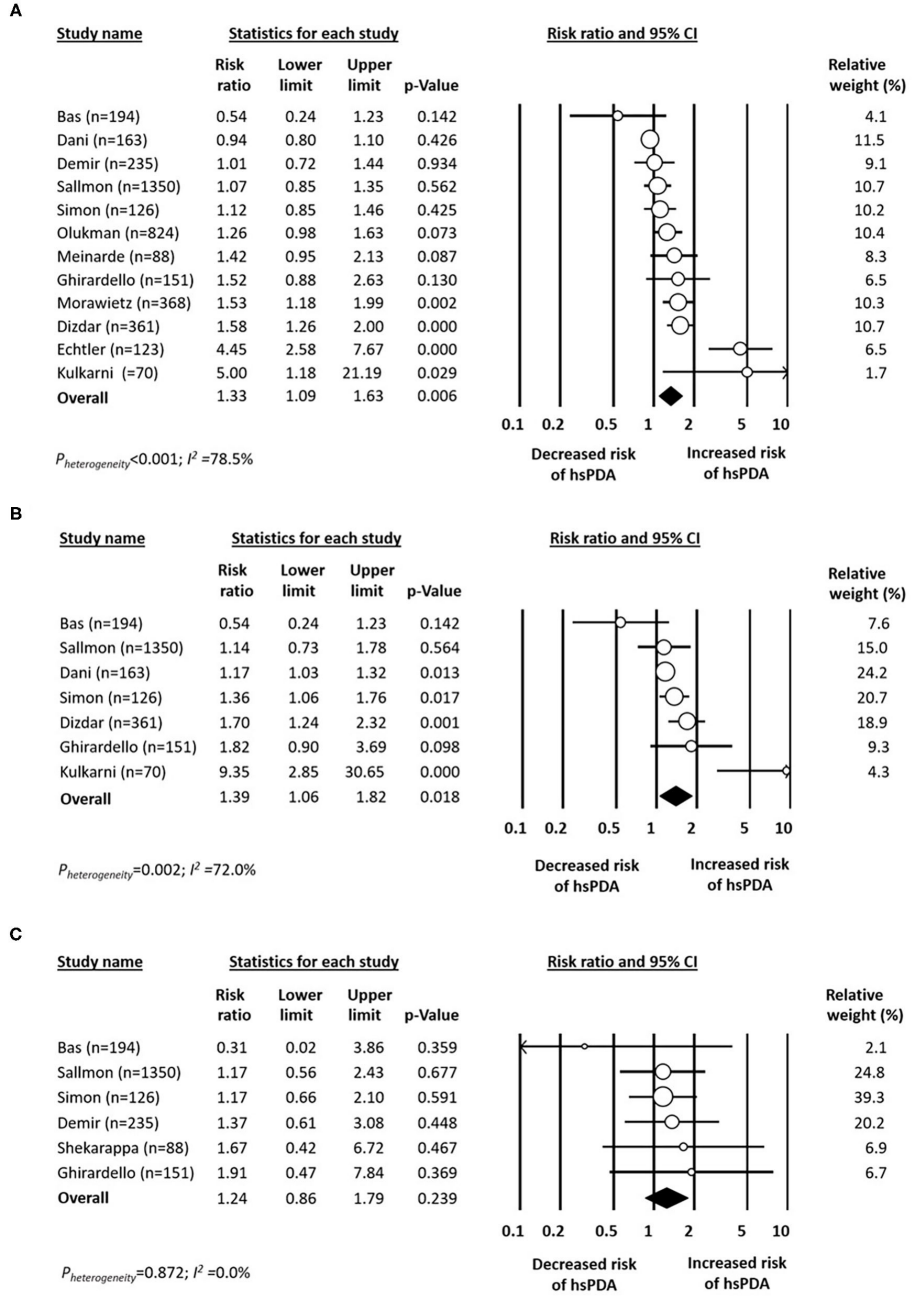
Forest plot for studies examining the association between platelet counts **(A)** below 150 × 10^9^/L, **(B)** below 100 × 10^9^/L, and **(C)** below 50 × 10^9^/L in the first day(s) of life on hemodynamically significant patent ductus arteriosus (hsPDA). Risk ratio >1 indicates increased risk of hsPDA.

Data on rate of hsPDA in infants with platelet counts above or below 150 × 10^9^/L were available from 12 studies (11–13, 16, 18, 19, 25, 28, 32–35). There was a significant positive association between hsPDA and platelet counts below 150 x 10^9^/L (Figure 3A). Neither visual inspection of the funnel plot (Supplementary Figure 2B) nor Egger's regression test (*P* = 0.130) revealed evidence of significant publication bias.

Data on rate of hsPDA in infants with platelet counts above or below 100 × 10^9^/L were available from seven studies (12, 13, 16, 18, 19, 28, 32). There was a significant positive association between any PDA and platelet counts below 100 x 10^9^/L (Figure 3B). Data on rate of hsPDA in infants with platelet counts above or below 50 × 10^9^/L were available from six studies (16, 18, 19, 25, 28, 36). Meta-analysis could not detect a significant association between any PDA and platelet counts below 50 × 10^9^/L (Figure 3C).

Mean (SD) platelet counts in infants with and without hsPDA were available from 19 studies (12, 13, 17–19, 22–31, 33, 35, 40, 41). Platelet counts were significantly higher in the group without hsPDA (Figure 2B). Neither visual inspection of the funnel plot (Supplementary Figure 2C) nor Egger's regression test (*P* = 0.941) revealed evidence of significant publication bias.

**Figure 4:**
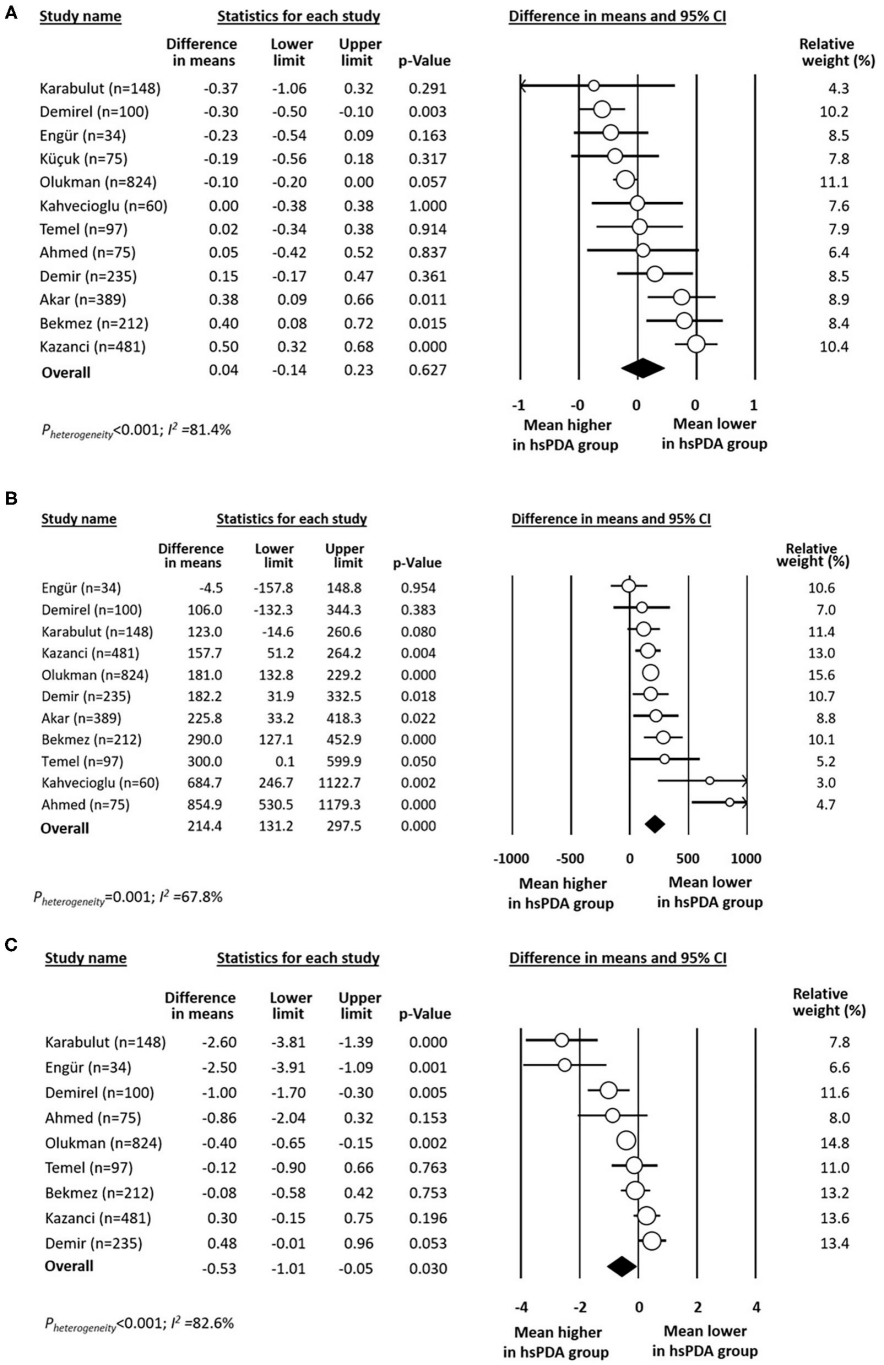
Forest plot for studies examining the difference (PDA-no minus PDA-yes) in **(A)** mean platelet volume (MPV), **(B)** platelet mass, and **(C)** platelet distribution width (PDW) in the first day(s) of life in infants with and without hemodynamically significant PDA (hsPDA).

Data on mean MPV in infants with and without any hsPDA were available from 12 studies (22–27, 29–31, 35, 40). Meta-analysis could not demonstrate a significant difference in MPV mean between the two groups (Figure 4A). Neither visual inspection of the funnel plot (Supplementary Figure 2D) nor Egger's regression test (*P* = 0.678) revealed evidence of significant publication bias.

Data on mean platelet mass in infants with and without any hsPDA were available from 11 studies (22–27, 29, 30, 35, 40, 41). Platelet mass was significantly higher in the group without hsPDA (Figure 4B). Neither visual inspection of the funnel plot (Supplementary Figure 2E) nor Egger's regression test (*P* = 0.212) revealed evidence of significant publication bias. Data on mean PDW in infants with and without any hsPDA were available from nine studies (22, 23, 25–27, 30, 35, 40, 41). Mean PDW was significantly lower in the hsPDA group (Figure 4C).

### Meta-Regression Analyses

Potential sources of heterogeneity were assessed through random effects (method of moments) meta-regression analysis (46). Meta-regression was conducted only for the meta-analyses including 10 or more studies. The results of the meta-regression analyses are summarized in Supplementary Table 4. Meta-regression showed that the mean (or median) GA and BW of the cohort significantly correlated with the effect size of the association between any PDA and platelet counts <150 × 10^9^/L (Supplementary Table 4 and Figure 2). The GA of the cohort was associated with 22% (*R*^2^ analog = 0.22) of the variance in the association between any PDA and platelet counts <150 × 10^9^/L across studies. Each week that the GA of the cohort increased resulted in an increase in PDA log RR of 0.16 (the equivalent of going from a RR of 1.00 to a RR of 1.45). Similarly, the BW of the cohort was associated with 15% (*R*^2^ analog = 0.15) of the variance in the association between any PDA and platelet counts <150 × 10^9^/L across studies. Each increment of 100 g in the BW of the cohort resulted in an increase in PDA log RR of 0.11 (the equivalent of going from a RR of 1.00 to a RR of 1.29).

Meta-regression also showed that the rate of hsPDA of the cohort significantly correlated with the effect size of the association between any hsPDA and platelet counts <150 × 10^9^/L (Supplementary Table 4 and Figure 2). The hsPDA rate of the cohort was associated with 17% (*R*^2^ analog = 0.17) of the variance in the association between hsPDA and platelet counts <150 × 10^9^/L across studies. Each 10% decrease in hsPDA rate resulted in an increase in hsPDA log RR of 0.12 (the equivalent of going from a RR of 1.00 to a RR of 1.32).

## Discussion

This updated meta-analysis included 21 additional studies and doubled the number of infants, providing stronger evidence of the association between low platelet counts in the first day(s) of life and the risk of developing PDA or hsPDA. Besides low platelet counts, other platelet parameters such as platelet mass and PDW showed a significant association with hsPDA risk. Although the meta-analysis has gained in robustness with the increase in the number of studies and the inclusion of other platelet parameters, our results are still limited by the marked clinical and statistical heterogeneity among the different studies. In addition, the question that remains unanswered is to what extent the low number of platelets is an epiphenomenon associated with the maturity and clinical stability of preterm infants rather than a contributing factor in the pathogenesis of PDA (15).

As already discussed in the previous version of the meta-analysis, one of the main limitations for the study of the association between thrombocytopenia and PDA is the absence of uniform criteria to establish when these two entities have a clinical relevance (19). It has been argued that the definition of thrombocytopenia based on thresholds of 150 or even 100 × 10^9^/L platelets is not clinically relevant for very preterm newborns (47, 48). In addition, neonatal thrombocytopenia and PDA share some potential etiopathogenic factors and this may confound the study of their potential association. The most frequent etiology of thrombocytopenia developing within the first days of life is intrauterine growth restriction (IUGR) and/or maternal hypertension (49, 50). This form of thrombocytopenia is rarely severe and platelet counts return to normal spontaneously in a short period of time (49, 50). The possible association between IUGR and PDA is controversial with studies reporting either a higher or lower risk of PDA in preterm infants with a history of IUGR (51). We analyzed this association in a recent meta-analysis (51). We observed a negative association between PDA and antenatal growth restriction (51). However, this association was only present in the meta-analysis pooling the studies that defined growth restriction as small for gestational age. The cohorts in which fetal growth assessment was conducted did not show a significant association with the risk of developing PDA (51). Therefore, the current evidence does not suggest that IUGR increases the incidence of PDA. Another pathological condition associated with both early-onset thrombocytopenia and PDA is perinatal infection (49, 50). Although individual studies and meta-analyses have shown an association between chorioamnionitis and PDA risk (52–54), this association was significantly biased by the lower GA of the group of infants exposed to chorioamnionitis (53). Nevertheless, neonatal sepsis is widely considered to be a major factor in the pathway leading from PDA to hsPDA (55).

Another important limitation of the previous (19) and present meta-analysis is the significant statistical heterogeneity observed in most of the analyses. We performed meta-regression analysis in order to investigate potential sources of heterogeneity. Meta-regression data suggest that the association between any PDA and platelet counts below 150 × 10^9^/L correlated with the GA and BW of each cohort. That is, those studies that included children with higher GA and BW showed a stronger association between thrombocytopenia and PDA. However, this finding could not be confirmed in the other meta-regression analyses that we performed. The incidence of both PDA and hsPDA was highly variable across the different studies (see Table 1). This variability is probably a consequence of the lack of consensus on clinical or sonographic criteria to consider a ductal shunt as significant (1–10). Meta-regression showed that the association between hsPDA and low platelet counts (<150 × 10^9^/L) was stronger in the studies with a lower rate of hsPDA. Nevertheless, our meta-regression results should be interpreted with caution. The minimum number of studies recommended to perform a meta-regression analysis is 10 (46). Our analyses were conducted in most cases with 10–11 studies but a higher number would have been necessary to obtain more reliable results.

Several investigators proposed that it is not the number of platelets but other quantitative or qualitative platelet parameters that are associated with a greater risk of developing PDA. Since large platelets are more reactive than small platelets, an elevated platelet size (i.e., a high MPV) is considered a marker of platelet activation (56, 57). Elevated MPV has been associated with cardiovascular disease in adults (58) and several studies have analyzed the association between MPV and complications of prematurity such as bronchopulmonary dysplasia, sepsis, intraventricular hemorrhage, retinopathy of prematurity, or PDA (59–63). Nevertheless, our meta-analysis could not demonstrate an association between MPV and hsPDA (Figure 4). In contrast, the platelet mass was significantly lower in the group of infants with hsPDA. The platelet mass and the plateletcrit are calculations obtained by multiplying the MPV by the platelet count (44, 45). For example, a platelet count of 100 × 10^9^/L and a MPV of 10 fL would equate to a platelet mass of 1,000 (45). Since in the mathematical formula the platelet count has a factor of hundreds while the MPV has a factor of tens, the former will have a weight 10 times greater than the latter in the final value of the platelet mass. Therefore, our findings regarding the association of platelet mass with hsPDA may be only a repetition of the findings on the association of platelet counts and risk of hsPDA.

As mentioned above, large platelets are considered to be metabolically and enzymatically more active than small platelets. PDW reflects the variability in the platelet size and its increase is considered as a sign of platelet activation (56, 57). According to the hypothesis of the role of platelets in DA closure, a higher degree of platelet activation, and therefore a higher PDW, would be expected in the infants without PDA. However, our meta-analysis shows that infants with hsPDA have higher PDW values than infants without the condition. The heterogeneity of this meta-analysis was very high and the included studies showed either increased or reduced PDW in the hsPDA group. In addition, high PDW and MPV have been proposed as useful markers to distinguish thrombocytopenia associated with sepsis from other etiologies (56). Thus again, the etiology of thrombocytopenia (i.e., sepsis vs. IUGR) may be the factor leading to the differences in results of the studies addressing the association between platelet parameters and PDA.

The hypothesis that the important risk factor for PDA is altered platelet function, not decreased platelet number, has also been investigated. Unfortunately, the low number of studies that included information on this topic did not allow us to pool them in a meta-analysis. Immature platelets may have different functional features when compared to mature platelets (64). Sallmon et al. investigated the immature platelet fraction in a cohort of infants with and without hsPDA (64). Although higher immature platelet fraction values were associated with hsPDA, logistic regression analysis revealed that only mature platelet counts on postnatal day 7 were independently associated with the condition (64). Ghirardello et al. investigated thromboelastographic, a technique for bedside hemostatic assessment, in preterm infants with and without hsPDA (28). They could not demonstrate significant differences in thromboelastographic profile in children having hsPDA, or in those who failed pharmacological treatment, compared to their respective controls (28). Engur et al. (27) and Ahmed et al. (23) reported significant lower levels of platelet derived growth factor in the first days of life in preterm infants developing hsPDA. Finally, Kahvecioglou et al. reported that a higher collagen-ADP duration was an independent risk factor for hsPDA. All these findings need to be confirmed in future studies.

It is still unclear which long-term benefits or harms are achieved by treating a PDA (1–9). The attitude of neonatologists toward PDA has progressively changed in the last years from advocating widespread treatment to close the PDA to a call for watchful observation (1–9). Although our meta-analysis focused on the effect of thrombocytopenia on “spontaneous” DA closure, we must take into account that rate of PDA closure may have been affected by the variations in the management of PDA among centers (8, 9). In a recent meta-analysis, Mitra et al. reported that low platelet counts were associated with higher odds of failure to PDA treatment with ibuprofen or indomethacin (65). Therefore, taking together the results of the meta-analysis of Mitra et al. and the present one, the evidence points to an association between low platelet counts and the presence of PDA as well as the lack of response to pharmacological treatment. Nevertheless, association does not mean causation.

Besides the observational studies on the association between platelet counts and PDA, the potential effect of platelet transfusion on DA closure has been investigated in two randomized controlled trials (RCTs). Andrew et al. compared two platelet count thresholds for prophylactic platelet transfusion (150 × 10^9^/L vs. 50 × 10^9^/L) (66). The incidence of either the primary outcome, intraventricular hemorrhage, or PDA, which was one of the secondary outcomes, was not significantly different between the two thresholds. (66). Very recently, Kumar et al. conducted a RCT with PDA as primary outcome and prophylactic transfusion thresholds of 100 × 10^9^/L vs. 20 × 10^9^/L platelets (67). This RCT could not demonstrate any positive effect of the higher threshold on DA closure. Interestingly, the group of infants in the higher threshold had a higher incidence of intraventricular hemorrhage (67). As reviewed by Fustolo-Gunnink et al., results from several studies suggest that platelet transfusions do not reduce bleeding risk of preterm infants, and even might increase it (68). Moreover, a recent RCT, which included 660 preterm infants (GA < 34 weeks), reported a risk reduction of major bleeding and/or mortality in neonates assigned to a prophylactic platelet transfusion threshold of 25 × 10^9^/L vs. a threshold of 50 × 10^9^/L. These results have led to a revision of the criteria and guidelines for platelet transfusion in very preterm infants to be more restrictive (68–70). Altogether, the current evidence suggests that increasing platelet counts by transfusion to hypothetically accelerate DA closure may generate more harm than benefit.

In conclusion, the present updated meta-analysis provides stronger evidence of the association between low platelet counts in the first day(s) of life and risk of developing PDA and hsPDA. Identifying simple markers of platelet function may improve our diagnostic and therapeutic decisions in dealing with PDA or other neonatal conditions. However, due to the complexities involved with the pathogenesis of both neonatal thrombocytopenia and PDA, it is impossible to establish a cause-and-effect relationship between the two conditions. In fact, studies that have attempted to correct the thrombocytopenia through platelet transfusions have not only failed to alter the incidence of PDA, but also have even led to increased morbidity (67).

## Data Availability Statement

The original contributions presented in the study are included in the article/Supplementary Material, further inquiries can be directed to the corresponding author/s.

## Author Contributions

GG-L carried out the systematic search, selected studies for inclusion, extracted study data, contributed to statistical analyses, interpretation of results, and drafting of the manuscript. SG supervised study inclusion and data collection, contributed to interpretation of results, and reviewed and revised the manuscript. PB-S contributed to search and data collection, contributed to interpretation of results, and reviewed and revised the manuscript. GC supervised study inclusion and data collection, contributed to interpretation of results, and reviewed and revised the manuscript. FM and RC contributed to interpretation of results, and reviewed and revised the manuscript. EV conceptualized the study, carried out the systematic search, selected studies for inclusion, extracted study data, carried out the statistical analyses and interpretation of results, and drafted the manuscript. All authors contributed to the article and approved the submitted version.

## Conflict of Interest

The authors declare that the research was conducted in the absence of any commercial or financial relationships that could be construed as a potential conflict of interest.
