# Corrigendum: Platelet Counts and Patent Ductus Arteriosus in Preterm Infants: An Updated Systematic Review and Meta-Analysis

**DOI:** 10.3389/fped.2021.694606

**Published:** 2021-05-19

**Authors:** Gema González-Luis, Stefano Ghirardello, Pilar Bas-Suárez, Giacomo Cavallaro, Fabio Mosca, Ronald I. Clyman, Eduardo Villamor

**Affiliations:** ^1^Department of Neonatology, Complejo Hospitalario Universitario Insular Materno-Infantil (CHUIMI) de Canarias, Las Palmas de Gran Canaria, Spain; ^2^Neonatal Intensive Care Unit, Fondazione IRCCS Ca' Granda Ospedale Maggiore Policlinico, Milan, Italy; ^3^Department of Pediatrics, Hospital Vithas Santa Catalina, Las Palmas de Gran Canaria, Spain; ^4^Department of Clinical Sciences and Community Health, Università degli Studi di Milano, Milan, Italy; ^5^Cardiovascular Research Institute, Department of Pediatrics, University of California, San Francisco, San Francisco, CA, United States; ^6^Department of Pediatrics, Maastricht University Medical Center (MUMC+), School for Oncology and Developmental Biology (GROW), Maastricht, Netherlands

**Keywords:** ductus arteriosus, platelets, meta-analysis, platelet distribution width, thrombocytopenia

An author name was incorrectly spelled as **Stefano Ghiradello**. The correct spelling is **Stefano Ghirardello**.

In the published article, there was an error in affiliation **2**. Instead of “**Neonatal Intensive Care Unit, Department of Clinical Sciences and Community Health, Fondazione IRCCS Cà Granda Ospedale Maggiore Policlinico, Università degli Studi di Milano, Milan, Italy**.”, it should be “**Neonatal Intensive Care Unit, Fondazione IRCCS Ca' Granda Ospedale Maggiore Policlinico, Milan, Italy**.”

In the published article, there was an error regarding the affiliation for **Fabio Mosca**. As well as having affiliation **2**, they should also have **Department of Clinical Sciences and Community Health, Università degli Studi di Milano, Milan, Italy**.

The authors apologize for this error and state that this does not change the scientific conclusions of the article in any way. The original article has been updated.

