# Supplementary material for: Platelet Counts and Patent Ductus Arteriosus in Preterm Infants: An Updated Systematic Review and Meta-Analysis
Source: Front Pediatr. 2021 Jan 20;8:613766. doi: 10.3389/fped.2020.613766 (PMC7854898; doi:10.3389/fped.2020.613766)
Supplement: Supplementary file 1 [file Data_Sheet_1.PDF]

Platelet counts and patent ductus arteriosus in preterm infants: an updated systematic review and meta-analysis

Gema González-Luis[1], Stefano Ghiradello[2], Pilar Bas-Suárez[3], Giacomo Cavallaro[2], Fabio Mosca[2], Ronald I Clyman[4], Eduardo Villamor[5*].

[1]Neonatal Service, Hospital Universitario Materno-Infantil de Canarias, Las Palmas de Gran Canaria, Spain.

[2]Neonatal Intensive Care Unit, Department of Clinical Sciences and Community Health, Fondazione IRCCS Cà Granda Ospedale Maggiore Policlinico, Università degli Studi di Milano, Milan, Italy.

[3]Department of Pediatrics, Hospital Vithas Santa Catalina, Las Palmas de Gran Canaria, Spain.

[4]Cardiovascular Research Institute and Department of Pediatrics, University of California San Francisco, San Francisco, California, USA.

[5]Department of Pediatrics, Maastricht University Medical Center (MUMC+), School for Oncology and Developmental Biology (GROW), Maastricht, the Netherlands.

*** Correspondence:**
Eduardo Villamor
e.villamor@mumc.nl

Supplementary Material

Supplementary Table 1. Methodological quality assessment through the Newcastle-Ottawa Scale (NOS)

First author, year	Selection	Comparability	Outcome/ Exposure	Total
Echtler, 2010	****	**	***	9
Fujioka, 2011	***	*	***	7
Shah, 2011	***	**	***	8
Dwarakanath, 2011	***	*	***	7
Dizdar, 2012	***	**	***	8
Sallmon, 2012	****	**	***	9
Dani, 2012	***	*	***	7
Brunner, 2013	***	**	***	8
Bas, 2014	***	*	***	7
Chen, 2014	***	*	***	7
Simon, 2015	***	*	***	7
Engür, 2015	***	*	***	7
Demir, 2016	****	*	***	8
Kulkarni, 2016	***	*	***	7
Meinarde, 2016	***	*	***	7
Morawietz, 2016	****	*	***	8
Oliveira, 2016	****	*	***	8
Olukman, 2016	****	**	***	9
Temel, 2017	***	*	***	7
Kahvecioglu, 2018	***	*	***	7
Bekmez, 2018	***	**	***	8
Küçuk, 2018	***	*	***	7
Akar, 2019	****	*	***	8
Karabulut, 2019	***	*	***	7
Kazanci, 2019	****	**	***	9
Saldaña, 2019	****	**	***	9
Demirel, 2020	***	*	***	7
Shekharappa, 2020	***	*	***	7
Ahmed, 2020	***	*	***	7
Ghirardello, 2020	****	*	***	8
Kusuma, 2020	***	**	***	8

Supplementary Table 2. Criteria used in each study for the definition of PDA and/or hemodynamically significant PDA (hsPDA).

Study	Year	PDA / hsPDA definition
Echtler et al.	2010	The PDA was considered hemodynamically relevant if the end-diastolic flow in the celiac trunk was <0, or the atrium/aorta diameter ratio (A/Ao) was >1.5, if a cerebral steal phenomenon was present or if there were any clinical signs indicative of hemodynamic relevance. In all other cases, patent DAs were considered hemodynamically irrelevant (end-diastolic flow in the celiac trunk >0, A/Ao <1.5, no clinical signs indicative of hemodynamic relevance of the PDA).
Fujioka et al.	2011	Echocardiography: criteria similar to Echtler et al.
Shah et al.	2011	Echocardiography: criteria not specified
Dwarakanath et al.	2011	Echocardiography: criteria not specified
Dizdar et al.	2012	Hemodynamically significant PDA based on echocardiographical findings was defined as one with an internal ductal diameter of ≥1.5 mm and/or with a left atrium (LA)/aortic root (AO) ratio ≥1.5.
Sallmon et al.	2012	A PDA with left to-right shunt was considered hemodynamically significant under the following conditions: (1) respiratory setback with supplemental inspiratory oxygen. 30% and/or mechanical invasive or noninvasive ventilation; (2) left atrium to aortic root ratio ≥ 1.4 by using M-mode; and/or (3) ductal diameter ≥ 2.5 mm; and/or (4) a decreased end diastolic flow in the anterior cerebral artery with a resistance index ≥ 0.85 in the cerebral ultrasound indicating significant ductal steal.
Dani et al.	2012	The diagnosis of hemodynamically significant PDA requiring treatment was made by echocardiographic demonstration of a ductal left- to-right shunt, with a left atrium-to-aortic root ratio >1.3 or a ductal size >1.5 mm (the minimal ductal size within the course of the ductus was measured).
Brunner et al.	2013	PDA was diagnosed by echocardiography. Echocardiography was routinely performed on day of life (DOL) 2 in infants who had severe RDS and on DOL 4-7 in those who were not ventilated, and treatment, if indicated, was started immediately after diagnosis of PDA.
Bas et al.	2014	hsPDA was defined by a transductal diameter >1.5 mm with unrestrictive (<1 m/s) left-to-right transductal flow on pulse wave Doppler and clinical signs of pulmonary overcirculation (e.g. increasing ventilation, oxygenation problems) and/or systemic hypoperfusion (e.g. abdominal distension, oliguria). In the absence of clear clinical signs, ascertainment of hemodynamic significance was made on the basis of any of the following echocardiographic characteristics: left atrial/aortic root ratio >1.4, mean velocity in the left pulmonary artery >0.6 m/s or diastolic backflow in the abdominal aorta.
Chen et al.	2014	PDA was defined by echocardiographic finding with clinical signs, and by need for treatment with drugs or surgery.
Simon et al.	2015	Patent ductus arteriosus (PDA) was defined as a requirement for indomethacin or ibuprofen and/or surgical ligation.
Engür et al.	2015	Infants with ductal diameters >1.5 mm and left atrium-to-aortic root ratios >1.5 with retrograde diastolic flow in the aorta and diastolic flow in the pulmonary artery >0.20 m/sec on day 5 were classified as “hemodynamically significant patent ductus arteriosus”
Demir et al.	2016	The 2D echocardiography criteria for hPDA were: a DA diameter of ≥ 1.4mm and/or the ratio of left atrium/aortic root of ≥ 1.4, and enlargement of the left ventricle.
Kulkarni et al.	2016	We defined PDA as any detectable blood flow across the DA by color Doppler....We scored the above parameters and used a composite score of ≥ 21 to define hsPDA, as per published validated criteria (Eur J Pediatr. 2013;172:179–1841)

Study	Year	PDA / hsPDA definition
Meinarde et al.	2016	Clinically significant PDA was defined by a transductal diameter ≥ 1.5 mm and left atrium to aortic ratio ≥1.5, with unrestrictive (<1 m/s) left-to-right transductal flow on pulse wave Doppler and clinical signs of pulmonary overcirculation (increasing ventilation, and oxygenation problems)
Morawietz et al.	2016	A hemodynamic relevant PDA was defined as such when one of the left-right shunt. The following parameters that could be verified by echocardiographic examinations were present: LA / Ao ratio> 1.4; ductus diameter> 2 mm at the narrowest point; a diastolic retrograde flow in the descending aorta.
Oliveira et al.	2016	Hemodynamically significant PDA was diagnosed as ductal diameter > 1.5 mm, relationship left atrium / aorta diameter > 1.4.
Olukman et al.	2016	hsPDA was defined as an internal ductal diameter of at least 1.5mm and LA/Ao of at least 1.4. Signs of clinical significance were accepted as the presence of continuous murmur, hyperactive precordium, tachycardia, left ventricular hypertrophy in electrocardiography, features of systemic hypoperfusion (e.g. low diastolic blood pressure, low systemic blood pressure, widened pulse pressure, bounding pulse, metabolic acidosis), features of pulmonary overcirculation (e.g. tachypnea, respiratory distress, increased oxygen or ventilation requirements, increased pulmonary vascular markings, and cardiomegaly on chest X-ray), and presence of apnea.
Temel et al.	2017	Hemodynamically significant PDA was defined as one with an internal ductal diameter of ≥1.5 mm and/or with a left atrium/aortic root ratio ≥1.5 echocardiographically.
Kahvecioglu et al.	2018	"Open PDA" was defined as the ductus with an internal diameter >1.5 mm and/or with a left atrium (LA)/aortic root (Ao) ratio >1.5.
Bekmez et al.	2018	The markers indicating hsPDA were the presence of ductal diameter ≥1.5 mm and/or a left atrial-to-aortic root (LA:Ao) ratio ≥1.5.
Küçuk et al.	2018	The diagnosis of PDA was made echocardiographically. PDAs with an internal diameter above 1.5 mm on colored Doppler evaluation and/or with a left atrium to aortic root ratio above 1.5 was deemed to be hemodynamically signicant.
Akar et al.	2019	Hemodynamically significant PDA was diagnosed with echocardiographic examination. Based on echocardiographic findings, hemodynamically significant PDA was defined as one with an internal ductal diameter ≥1.5 mm and/ or with a left atrium (LA)/aortic root (AO) ratio ≥1.5.
Karabulut et al.	2019	Preterm infants with at least 1 of the following clinical signs of PDA (hyperdynamic precordium, continuous murmur, tachycardia, hypotension, oliguria, increase in pulse pressure, increase in tension and/or oxygen need) and at least 1 of the following echocardiography findings (ductal diameter ≥ 1.5mm, left atrium/aorta root ratio ≥ 1.5, and insufficient diastolic flow or reverse flow in the abdominal aorta) were categorized as infants with hsPDA and were treated for DA closure.
Kazanci et al.	2019	hsPDA was defined as having a ductal diameter of ≥ 1.5 mm and/or left atrium/aortic root ratio of ≥ 1.5. Clinically significant findings of PDA were defined as continuousmurmur, tachycardia, hyperactive precordium, signs of systemic hypoperfusion (systolic/diastolic hypotension, wide pulse pressure, bounding pulses, metabolic acidosis, oliguria), and signs of pulmonary overcirculation (tachypnea, increased need for oxygen and ventilatory support, increased pulmonary vascular signs, and apnea).
Saldaña et al.	2019	Considering that newborns with absent or mild pulmonary hypertension represent the cases of non-hemodynamically significant PDA (nohsPDA); and those with moderate and severe pulmonary hypertension as hemodynamically significant PDA (hsPDA).
Demirel et al.	2020	hsPDA was defined as internal ductal diameter of ≥ 1.5mm and/or with a left atrium (LA)/aortic root (AO) ratio >1.5. Signs of clinical significant PDA were presence of continuous murmur, tachycardia, hyperactive precordium, features of systemic hypoperfusion as low

Study	Year	PDA / hsPDA definition
		systemic blood pressure, low diastolic blood pressure, widened pulse pressure, metabolic acidosis and features of pulmonary hyperperfusion as tachypnea, respiratory distress, increased oxygen, or ventilation requirements.
Shekharappa et al.	2020	PDA was classified, based on echocardiography, as small with PDA size <1.5 mm, left atrium (LA) and aortic valve (Ao) ratio <1.4:1, moderate with PDA size >1.5–3 mm, and LA: Ao >1.4:1 and large PDA size >3 mm with LA: Ao >1.6:1. hsPDA was considered, when size was >1.4 mm/kg or LA:Ao >1.4:1.
Ahmed et al.	2020	The Echocardiographic criteria used for considering PDA as hemodynamically significant PDA (HDsPDA) include: Infants with ductal diameters >1.5 mm/ kg, left atrium to aortic root ratios >1.4 with retrograde diastolic flow in the aorta and diastolic flow in the pulmonary artery >0.2 m/sec were classified as HDsPDA.
Ghirardello et al.	2020	The hsPDA was defined as a DA with an internal diameter ≥1.5 mm with exclusive left-to-right shunt, a left atrium/aortic root ratio ≥1.5, and evidence of pulmonary overcirculation or systemic shunt effect, according to McNamara et al. (Arch Dis Child Fetal Neonatal Ed. 2007; 92:F424–7)
Kusuma et al.	2020	The inclusion criteria in this study including: preterm infants (gestational age <37 weeks), parents agreed to participate in the study (proven by the signing of the informed consent) and suffered from isolated PDA from echocardiographic examination (specifically for case groups).

Supplementary Table 3. Sensitivity analyses excluding one study at a time

PDA type	Meta-analysis	Removed Study	Risk ratio (RR) or difference in means (DM) (95% CI interval)
Any PDA	Platelets < 150 x 10^9/L	None	RR 1.58 (1.28–1.95)
		Saldaña et al.	RR 1.44 (1.20–1.72)
		Sallmon et al.	RR 1.67 (1.34–2.08)
	Platelets < 100 x 10^9/L	None	RR 1.61 (1.14-2.28)
		Kulkani et al.	RR 1.39 (1.05–1.85)
		Shah et al.	RR 1.82 (1.29–1.85)
	Platelets < 50 x 10^9/L	None	RR 1.34 (0.77–2.32)
		Saldaña et al.	RR 1.07 (0.79–1.46)
		Shah et al.	RR 1.47 (0.81–2.64)
Hemodynamically significant PDA	Platelets < 150 x 10^9/L	None	RR 1.33 (1.09–1.63)
		Echtler et al.	RR 1.22 (1.04–1.43)
		Dani et al.	RR 1.39 (1.13–1.72)
	Platelets < 100 x 10^9/L	None	RR 1.39 (1.06–1.82)
		Kulkarni et al.	RR 1.29 (1.06–1.57)
		Dani et al.	RR 1.49 (1.02–2.17)
	Platelets < 50 x 10^9/L	None	RR 1.24 (0.86–1.79)
		Ghirardello et al.	RR 1.21 (0.83–1.76)
		Simon et al.	RR 1.29 (0.81–2.07)
	Mean platelet counts	None	DM 22.0 (14.9–29.1)
		Chen et al.	DM 23.4 (16.7–30.0)
		Karabulut et al.	DM 20.2 (13.2–27.3)
	Mean platelet volume (MPV)	None	DM 0.045 (-0.136–0.226)
		Kazanci et al.	DM -0.010 (-0.156–0.136)
		Demirel et al.	DM 0.085 (-0.102–0.272)
	Platelet mass	None	DM 214.4 (131.2–297.5)
		Ahmed et al.	DM 174.3 (115.6–233.1)
		Engur et al.	DM 236.7 (153.0–320.4)
	Platelet distribution width (PDW)	None	DM -0.531 (-1.010 to -0.052)
		Demir et al.	DM -0.678 (-1.181 to -0.175)
		Karubulut et al.	DM -0.321 (-0.748–0.106)

The table shows the range of effect sizes after removing the study. Example: in the meta-analysis of the association between any PDA and platelets <150 x 10^9/L, the summary RR ranged from 1.44 (95% CI 1.20–1.72), when the study of Saldaña et al. was excluded, to 1.67 (95% CI 1.34–2.08), when the study of Sallmon et al. was excluded.

Supplementary Table 4. Meta-regression analyses

Association	Covariate	Number of studies	Coefficient	95% CI		P	R² analog
				Lower limit	Upper limit		
Difference in mean platelet counts and hsPDA	% hsPDA in cohort	19	-0.352	-0.941	0.238	0.243	0.0
	n of the study	19	-0.003	-0.039	0.033	0.871	0.0
	GA of cohort	19	5.343	-1.535	12.222	1.12	0.0
	BW of cohort	18	0.003	-0.035	0.041	0.877	0.0
	% males	17	-1.935	-3.628	-0.241	0.025	0.07
Platelet counts <150 x 10^9/L and any PDA	% PDA in cohort	11	-0.011	-0.024	0.003	0.124	0.0
	n of the study	11	-0.0005	-0.001	0.000	0.642	0.14
	GA of cohort	10	0.161	0.058	0.264	0.002	0.22
	BW of cohort	10	0.001	0.000	0.002	0.012	0.15
	% males	11	-0.048	-0.147	0.050	0.338	0.0
	% PL<150 x 10^9/L	11	0.003	-0.019	0.024	0.803	0.0
Platelet counts <150 x 10^9/L and hsPDA	% hsPDA in cohort	12	-0.011	-0.021	-0.002	0.019	0.18
	n of the study	12	-0.000	-0.001	0.000	0.434	0.0
	GA of cohort	12	0.163	-0.097	0.424	0.219	0.0
	BW of cohort	12	-0.000	-0.002	0.001	0.776	0.0
	% males	12	-0.044	-0.113	0.024	0.201	0.0
	% PL<150 x 109/L	12	0.009	-0.020	0.039	0.545	0.0
Difference in mean MPV and hsPDA	% hsPDA in cohort	12	-0.000	-0.020	0.019	0.973	0.0
	n of the study	12	0.000	-0.0005	0.001	0.417	0.0
	GA of cohort	12	-0.027	-0.174	0.120	0.719	0.01
	BW of cohort	11	-0.0001	-0.001	0.0007	0.739	0.0
	%males	11	0.007	-0.032	0.046	0.724	0.0
Difference in mean platelet mass and hsPDA	% hsPDA in cohort	11	0.556	-9.51	10.621	0.914	0.0
	n of the study	11	-0.125	-0.517	0.268	0.534	0.0
	GA of cohort	11	63.51	-17.232	144.265	0.123	0.0
	BW of cohort	10	0.036	-0.306	0.379	0.835	0.0
	% males	10	-11.577	-31.236	8.081	0.248	0.0

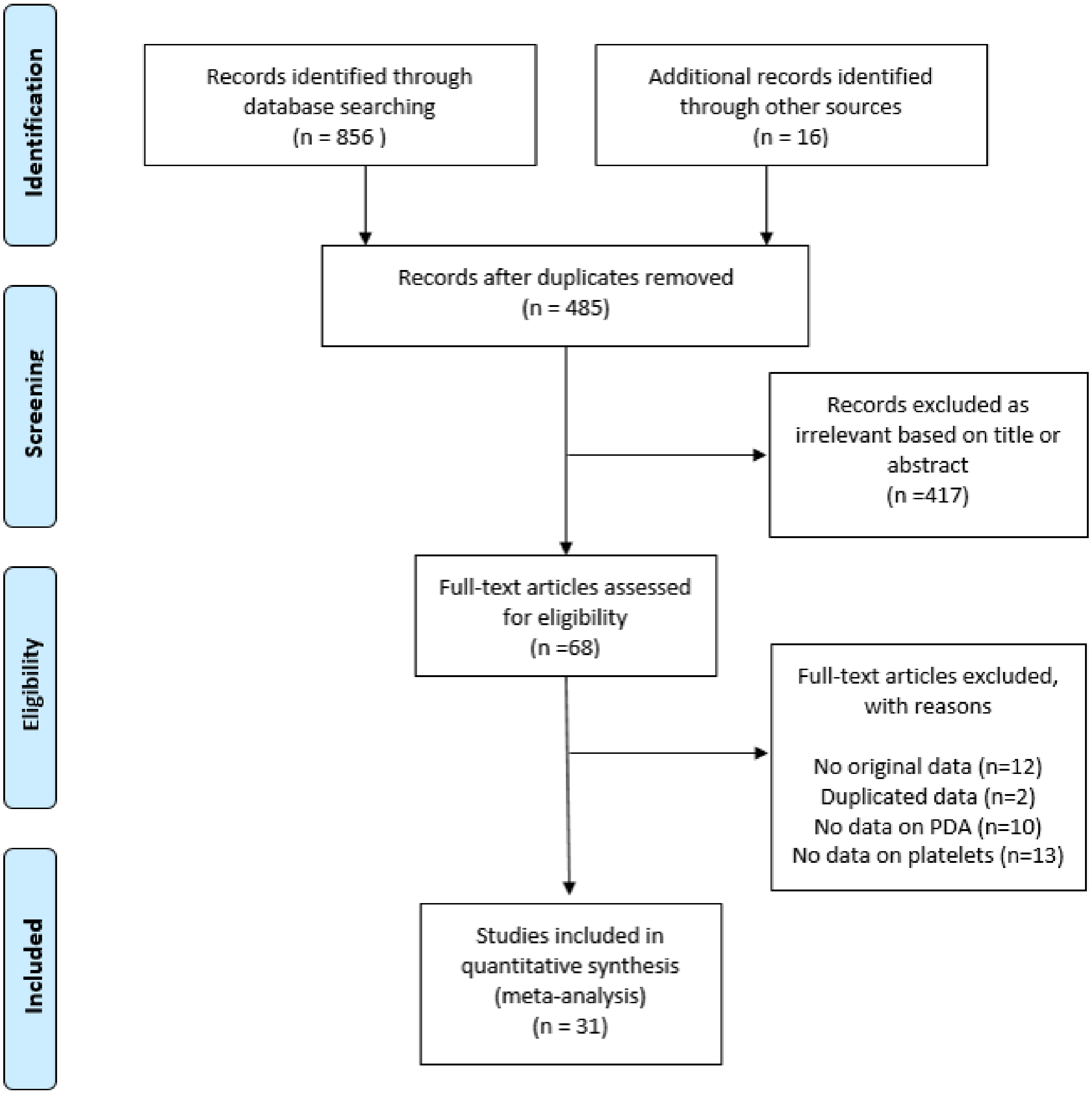

Supplementary Figure 1. PRISMA flow diagram of the search process.

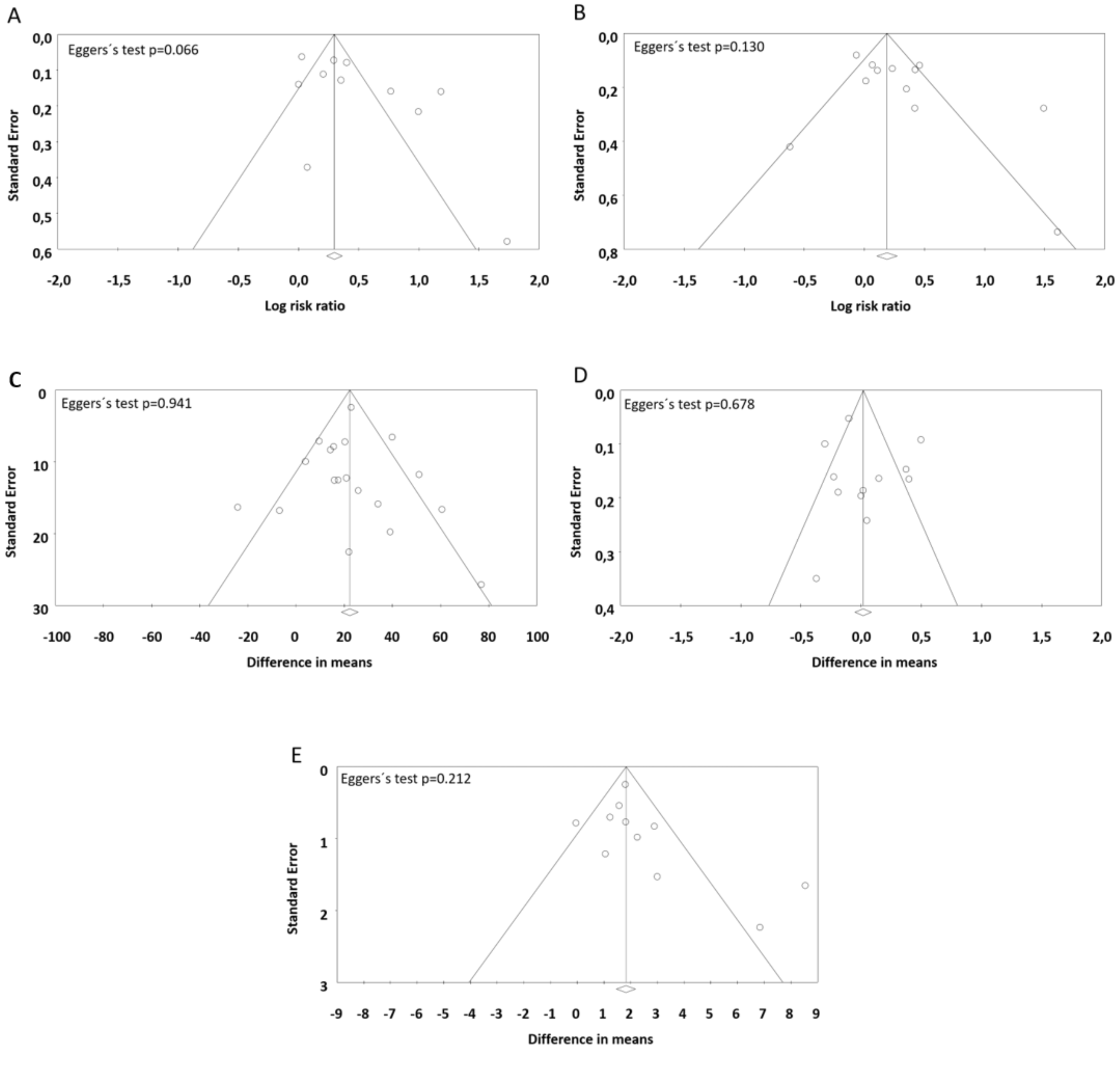

Supplementary Figure 2. Funnel plots for publication bias assessment.

Meta-analyses: A. Platelet counts < 150 x 10^9/L and any PDA; B. Platelet counts < 150 x 10^9/L and hemodynamically significant PDA (hsPDA); C. Difference in mean platelet counts and hsPDA; D. Difference in mean platelet volume (MPV) and hsPDA; E. Difference in mean platelet mass and hsPDA.

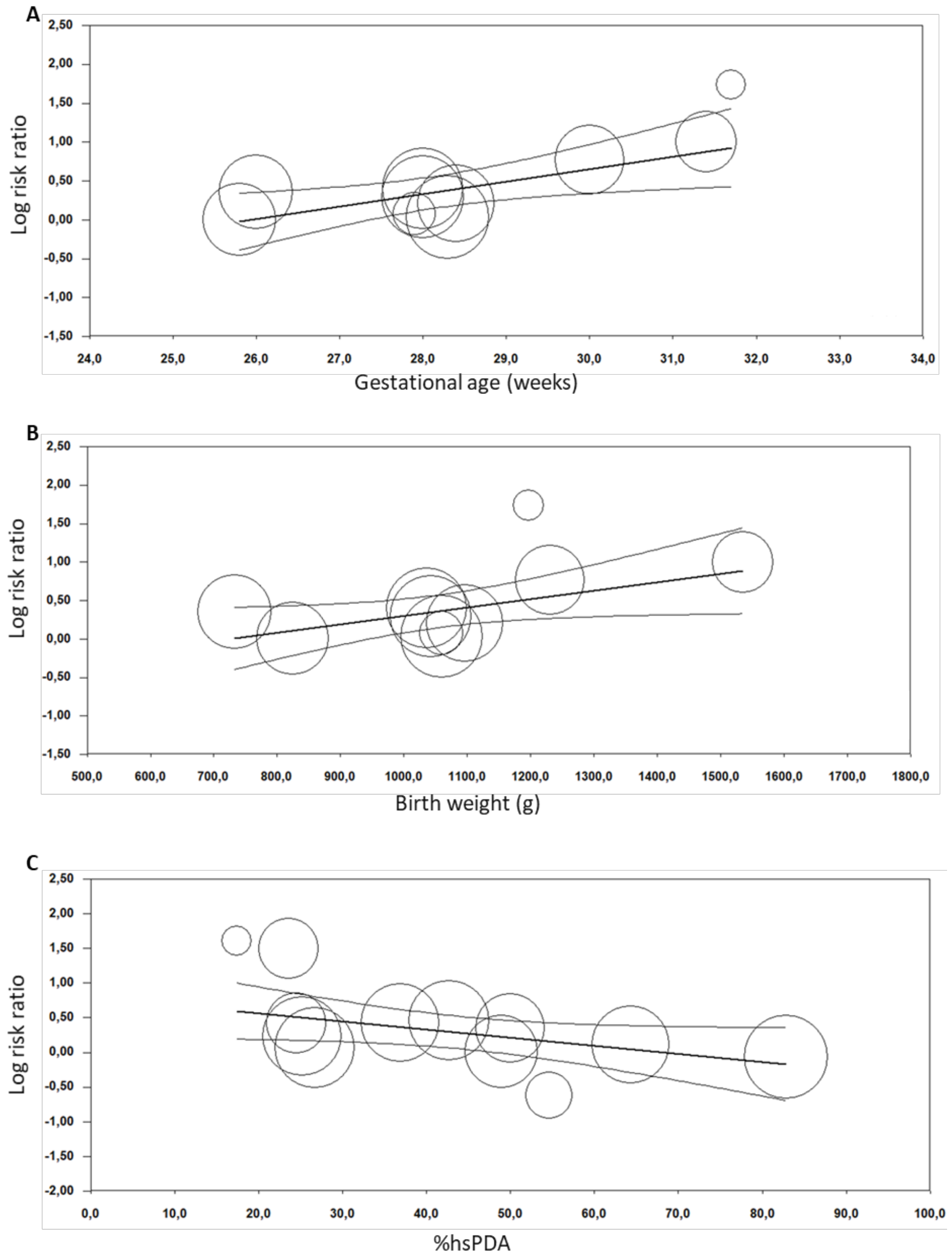

Supplementary Figure 3. A, B: Random-effects (methods of moments) meta-regression analysis of the correlation between birth weight (A) and gestational age (C) and risk (expressed as log risk ratio) of PDA with platelet counts below 150×10^9 /L in the first day(s) of life. **C:** Random-effects (methods of moments) meta-regression analysis of the correlation between rate of hemodynamically significant PDA (hsPDA) in the total cohort and risk (expressed as log risk ratio) of hsPDA with platelet counts below 150×10^9 /L in the first day(s) of life. Each circle represents an individual study.
